# The impact of cost-sharing on prescription drug demand: evidence from a double-difference regression kink design

**DOI:** 10.1007/s10198-022-01446-w

**Published:** 2022-02-25

**Authors:** Simona Gamba, Niklas Jakobsson, Mikael Svensson

**Affiliations:** 1 Department of Economics and Finance, Universitá Cattolica Del Sacro Cuore, Milan, Italy; 2 Karlstad Business School, Karlstad University, Karlstad, Sweden; 3 School of Public Health and Community Medicine, Institute of Medicine, University of Gothenburg, Box 463, 405 30 Gothenburg, Sweden

**Keywords:** Cost-sharing, Drug consumption, Health-care, Moral hazard, Regression kink design, Natural experiment, C33, D12, I11, I18

## Abstract

Pharmaceuticals represent the third-largest expenditure item in health care spending in the OECD countries, and cost growth is around 5% per year in many OECD countries. One possible way to contain the rise in pharmaceutical spending is the use of cost-sharing schemes that makes insured individuals directly bear parts of the cost of a drug. This study estimates the price sensitivity of demand for prescription drugs using data on all prescription drug purchases from a random sample of 400,000 Swedes followed from 2010 to 2013. We use a regression kink design (RKD) by exploiting the kinked Swedish cost-sharing scheme to assess the price elasticity. Further, since the cost-sharing scheme has changed over time, we also use a double-difference RKD to account for potential confounding nonlinearities around the kink. Our results indicate that the standard RKD results are biased and exaggerate the price sensitivity. Our preferred double-difference RKD specifications show no or minor price sensitivity (95% CI price elasticity from − 0.12 to 0.02). The results are similar in several sub-group analyses across age groups, sexes, and income quartiles.

**Supplementary Information:**

The online version contains supplementary material available at 10.1007/s10198-022-01446-w.

## Introduction

Pharmaceuticals represent the third-largest expenditure item in health care spending in the OECD countries. In (real) per capita terms, spending has on average risen by 20% for the OECD average over the last five years, by 26% in the U.S. and 40% in Germany [1]. Across the OECD, total pharmaceutical spending was around USD 600 billion in 2018 [1], and the adoption of new drugs targeting complex conditions is predicted to continue increasing costs in the coming years. This study uses data from the Swedish health-care system, where pharmaceutical spending has increased by about 25% in the last five years. The projection for coming years is that drug spending will continue to grow substantially above the GDP growth [2].

One possible way to contain the rise in pharmaceutical spending is to introduce or increase cost-sharing to reduce what is typically referred to as ex-post moral hazard [3–5], i.e., higher demand and use of drugs when insured from the cost. If there is any substantial moral hazard in pharmaceutical consumption, cost-sharing will likely increase efficiency. A downside of cost-sharing is that it may reduce compliance with effective drug prescriptions and lead to adverse health effects [6–8]. To understand the impact of cost-sharing policies, it is necessary to establish the demand effects (price sensitivity) of cost-sharing schemes. In this study, we address this question and estimate the impact of cost-sharing on prescription drug demand using a quasi-experimental approach that leverages the nonlinearity of the Swedish cost-sharing scheme.

A major difficulty in establishing the effect of cost-sharing on demand is that the cost-sharing level that patients face is typically not random and regression analyses where demand is regressed on cost-sharing (or the price) are likely to be confounded by selection issues. In a systematic review of the cost-sharing effects on drug demand, Luiza et al. [9] identified 32 studies of which most indicated that cost-sharing reduces drug demand. However, they also concluded that the certainty of evidence was low to very low, mainly because most studies were observational with a significant risk of bias.

Studies that have used empirical approaches that can credibly address the causal effect of cost-sharing include the early and influential RAND health insurance experiment [see, e.g., 10–12], which indicated an estimate of the price elasticity for health care between − 0.1 and − 0.2. More recently, experimental evidence from the Oregon Health Insurance Experiment that randomized access to Medicaid for previously non-eligible uninsured adults showed that access to Medicaid with no cost-sharing substantially increased utilization [13]. Studies adopting quasi-experimental approaches have obtained similar results, both when focusing on the elderly [8, 14] and the poor [e.g., 15]. However, in these contributions, the authors could not distinguish the effect of increased copayments for physician visits (which would reduce visits and, thus, physicians’ opportunities to prescribe drugs) from the impact of increased copayments for prescription drugs per se. Papers that have evaluated changes in cost-sharing for drugs without concurrent changes in the cost-sharing for visits include, e.g., García-Gómez et al. [16], which evaluated the introduction of a small copayment (€1 per prescription) in Spain using a before-and-after reform comparison and found a reduction in utilization of 6.4%. Another paper that specifically addressed the price elasticity of drug cost-sharing, and is most closely resembling what we do in this study, is the regression kink design (RKD) study by Simonsen et al. [17].^1^ They used the Danish policy rule that cost-sharing varies with patients’ total drug expenditures during a fiscal year. By assessing behavior near the cut-off points, where the cost-sharing changes (“kinks”), they estimated a price elasticity ranging from − 0.2 to − 0.7.

The present paper aims to add empirical evidence on the price elasticity of demand for prescription drugs. We do this by using a novel empirical approach for causal inference in this context. We use detailed and high-quality register data on prescribed drug consumption for 400,000 randomly selected adult Swedes observed from 2010 to 2013. As in Simonsen et al. [17], we rely on an RKD in which we exploit the fact that the cost-sharing scheme contains kinks related to patients’ total accumulated out-of-pocket expenditures. In addition to previous evidence, we implement a double-difference RKD (DD-RKD) by exploiting a policy reform in 2012 that shifted the kinks in the cost-sharing scheme so that higher total spending had to be reached before cost-sharing was introduced. As argued by Landais [18], the DD-RKD is an important added feature since the RKD design may be susceptible to bias caused by a smooth nonlinear relationship between the assignment variable and the outcome variable, which can spuriously be picked up as a kink [19, 20].

## Policy setting and data

### Prescription drug cost-sharing scheme

The Swedish health-care system, organized in 21 regions, is mainly financed by proportional regional income taxes, and to a lesser degree by national government grants and out-of-pocket payments. Provision of health care is predominantly public, but around 12% of tax-financed (mostly primary) health care is carried out by private providers. The regions reimburse the private providers in the same way as their public counterparts, and the same rules and copayments apply.

Drugs are provided as clinical drugs in a hospital setting or as prescription drugs. The prices and reimbursement of clinical drugs, which make up a smaller total cost than prescription drugs, are determined independently in each health-care region (although with some collaboration around reimbursements and prices). For prescription drugs, a producer applies for reimbursement of a market-approved drug to the Dental and Pharmaceutical Benefits Agency (TLV) for inclusion in the Swedish pharmaceutical benefits (subsidy) scheme at the manufacturer’s suggested retail price. If a drug is approved, it is sold at private pharmacies throughout the country at the fixed price agreed by the producer and TLV. The clinical benefits, the severity of the condition treated, and the cost-effectiveness of the drug are important factors for reimbursement [21].

**Fig. 1:**
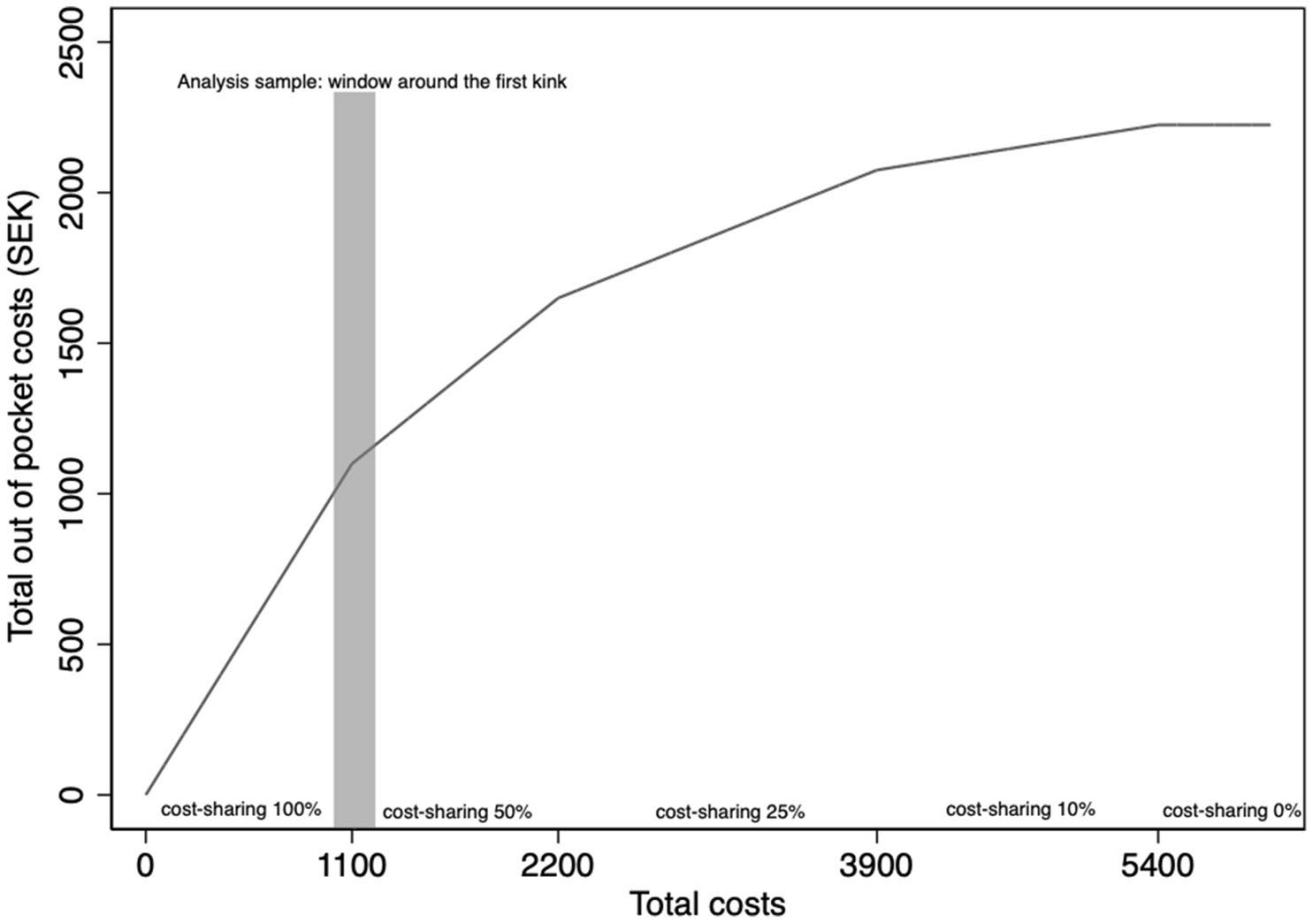
Out-of-pocket costs and total costs: cost-sharing scheme

The level of cost-sharing in the pharmaceutical benefits scheme is a continuous piecewise linear function of the accumulated cost of prescribed drugs purchased by the individual over a running 12-month period that we term the *fiscal year*. The subsidy scheme has been in place since 1997, and over this period, it has seen some changes to the cost thresholds. A relevant change for our analysis took place on January 1, 2012, and consisted of a 200-krona (SEK) increase (approx. $25 based on a SEK/USD exchange rate of 1 SEK = 0.125 USD) in all cost thresholds. The cost-sharing as a function of total accumulated prescription drug costs after the change in 2012 is shown in Fig. 1.

The first cost threshold (kink) is at SEK 1100 ($138), where cost-sharing goes from 100 to 50%, and this threshold will be used to identify the price elasticity of demand. Around 38% of individuals aged 18 and over incur total yearly costs equal to or higher than SEK 1100. The maximum out-of-pocket cost payable by an individual during the 12 months is SEK 2225 ($278), which applies to individuals with accumulated total costs exceeding SEK 5400 ($675).

### Data

We randomly sampled 400,000 individuals aged 18 and over living in Sweden in 2010 from Statistics Sweden’s Longitudinal Individual Database and retrieved data for these individuals up to 2013. The database contains demographic and economic information on age, area of residence, income, sex, educational attainment, employment status, etc. After accounting for mortality and emigration, the sample consists of 1,560,341 person-year observations.

Table A1 in the supplementary material shows summary statistics for the sample and the analysis sample (individuals who pass through the window around the first kink, as shown in Fig. 1). The mean age in our sample of 400,000 Swedes was 49, against 62 in the analysis sample. In the latter, the mean age was expected to be higher, given that this sample excludes, for example, individuals who have not purchased prescription drugs. The proportion of women is also higher in the analysis sample.

Subsequently, we merged the demographic and economic data with the Swedish National Prescribed Drug Register, which contains information on all individual purchases of prescribed drugs in Sweden (drug prices, prices paid by the consumers, purchase dates, etc.). Table A2 (supplementary material) shows summary statistics on prescription drug use based on the 1,560,341 person-year observations. In the analysis sample, mean total costs per year were SEK 4800 ($600), and mean out-of-pocket costs per year were SEK 938 ($117). The regional ethics committee in Sweden (Uppsala, #2014/270) approved the project and the data collection.

## Empirical methods

### Regression kink design

The regression kink design, utilizing the kinks in the cost-sharing scheme (Fig. 1), can be shown in a non-separable model [22]:

1 $$ Y = y\left( {B,V,U} \right), $$

where $Y$ is the probability of a prescribed drug being purchased, $B$ is the cost-sharing (price), $V$ is accumulated total pharmaceutical costs during the fiscal year, and $U$ is an error term. The challenge in estimating a parametric version of Eq. 1, in which *Y* is regressed on *B*, is that there are unobservables (such as the health status) related to both the subsidy (price) and the probability of a prescribed drug being purchased.

The regression kink design meets this challenge by using the relationship between *Y* and *B*, and *B* and *V*, respectively. Suppose the probability of a drug purchase ($Y$) depends on the cost-sharing ($B$), and the cost-sharing is a deterministic function of accumulated total costs ($V$). In that case, a kink in the relationship between *B* and *V* at a certain threshold should also cause a kink in the relationship between $Y$ and B [e.g., 22]. As outlined in Nielsen et al. [23] and, for example, by Card et al. [22], the constant-effect additive model:

2 $$ Y = \tau B + g\left( V \right) + u, $$

with $B=b(V)$ being a continuous and deterministic function of $V$ with a kink at $V=0$, and with the derivatives of $G(\cdot )$ and $E=\left[u|V=v\right]$ being continuous in $v$ at $v=0$, gives the RKD estimator ($\tau )$:

3 $$ \tau = \frac{{\mathop {\lim }\limits_{{v_{0} \to 0^{ + } }} \left. {\frac{dE\left| Y \right|V = v|}{{dv}}} \right|_{{v = v_{0} }} - \mathop {\lim }\limits_{{v_{0} \to 0^{ - } }} \left. {\frac{dE\left| Y \right|V = v|}{{dv}}} \right|_{{v = v_{0} }} }}{{\mathop {\lim }\limits_{{v_{0} \to 0^{ + } }} b^{\prime}\left( {v_{0} } \right) - \mathop {\lim }\limits_{{v_{0} \to 0^{ - } }} b^{\prime}\left( {v_{0} } \right)}}. $$

The numerator is the change in the slope of the probability of a drug purchase as a function of accumulated total costs at the kink while the denominator is the change in the slope of the cost-sharing at the kink. In a sharp RKD, the denominator is deterministic and equal to the change in the slope around the threshold (see Fig. 1).

Our analysis focuses only on the first kink where we have the best power (due to more observations); moreover, biases may be introduced if exploiting individuals in more than one kink during the same fiscal year [17]. For these reasons, we estimate the price elasticity around the SEK 1100 cost threshold where the denominator is equal to − 1/2. Our empirical approach relies on estimating the numerator in Eq. 3. The price elasticity ($\varepsilon$) can be computed by multiplying the RKD estimand by the sample mean price of the purchase ($\overline{\mathrm{price} }$) divided by the sample mean propensity to purchase another drug ($\overline{\mathrm{quantity} }$):

4 $$ \varepsilon = \tau \times \overline{{{\text{price}}}} /\overline{{{\text{quantity}}}} . $$

The parametric specification to retrieve the numerator in Eq. 3 is based on running parametric polynomials as:

5 $$ E\left[ {Y|V = v} \right] = \mu_{0} + \mathop \sum \limits_{p = 1}^{{\overline{p}}} \omega_{p} \left( {v - k} \right)^{p} + \beta_{p} \left( {v - k} \right)^{p} D + \theta X, $$

where $Y$ is an indicator for an individual making another prescription drug purchase in the fiscal year; $k$ is the kink; $v$, the running variable, is defined as the total cost of drugs purchased up to that point during the fiscal year; $D$ is an indicator for $v$ being above the kink. The parameter of interest, ${\beta }_{1}$, shows whether there is any change in the probability of a drug purchase at the kink. The vector $\mathbf{\rm X}$ contains a set of demographics, socioeconomic, and purchase-specific control variables. Of particular importance is a variable indicating the week of the fiscal year when the individual enters the bandwidth. This is important since it is likely to influence the ability to make another purchase during the fiscal year. In robustness checks, we also modify $Y$ to capture only any additional purchase in the following month, to restrict the time period for an additional purchase and make it equal for all individuals.

**Fig. 2:**
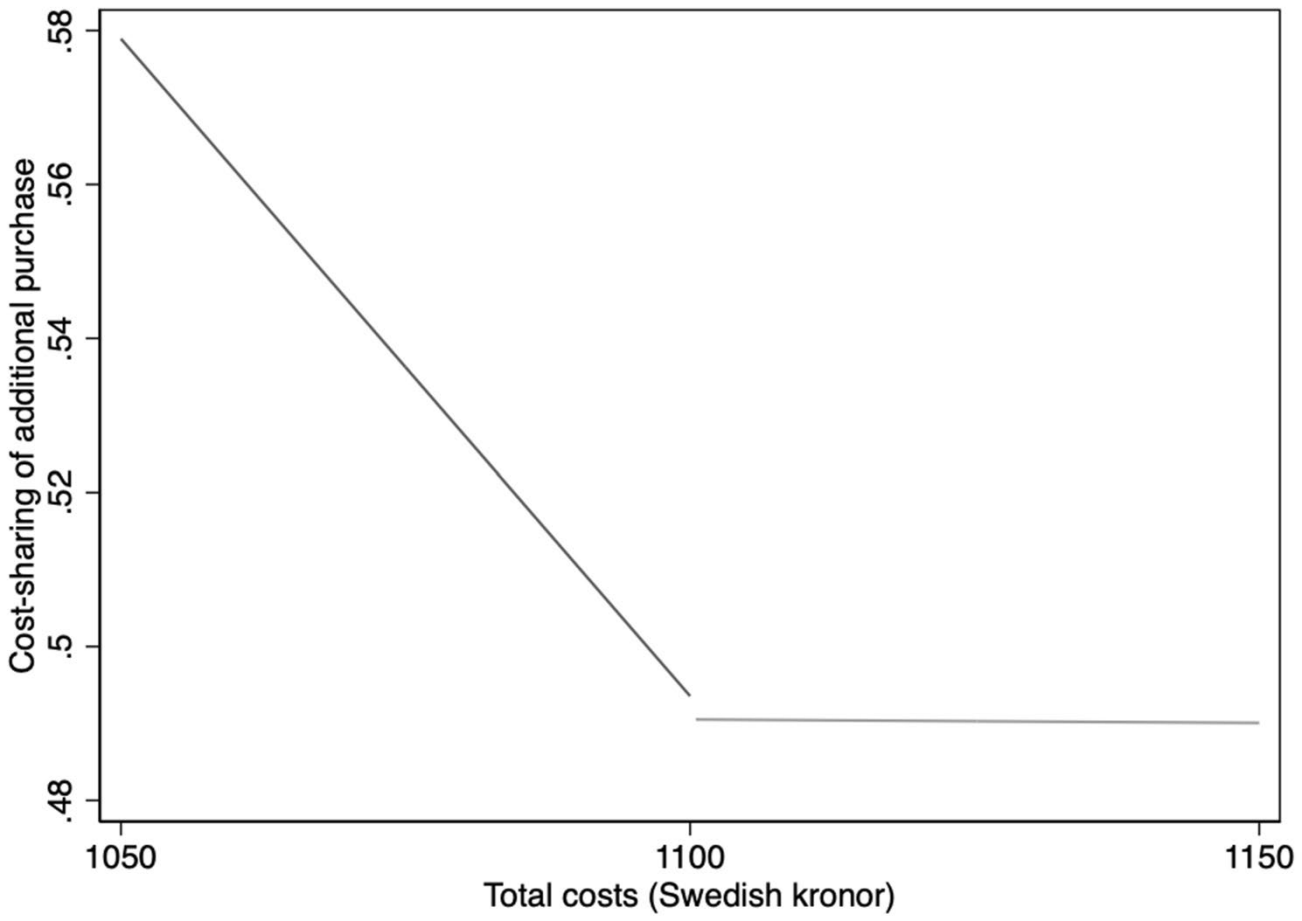
Total costs and share of patient cost

In estimating Eq. 5, we must choose the order of polynomials, the kernel, and the bandwidth. We follow the standard approach in the RKD literature and use first- and second-order polynomials while not considering cubic (third-order) or higher polynomials [24]. We use a uniform kernel [25], and in robustness analyses, we also consider a triangular kernel. As for the bandwidth choice, in Fig. 2, we show the patient cost-sharing (*B*) as a function of accumulated total costs (*V*) for our main bandwidth of SEK 50 ($6.25). Cost-sharing in the bandwidth to the left of threshold is on average 60% while cost-sharing in the bandwidth to the right of the threshold is 50%. There is a sharp kink at the threshold, which indicates that the data fulfill the fundamental assumption of a sharp kink at the SEK 1100 threshold.^2^ Further, we also consider all possible bandwidths in one SEK increment from 5 to 50 kronor, which is reported as part of our robustness checks.^3^

### Identifying assumptions

A potential threat to identifying a causal effect in an RKD is manipulation around the kink in terms of the distribution of observations and covariates. Figure A2 in the Supplementary material shows the frequency distribution of observations and covariate values in a 50 SEK bandwidth. More formally, on performing a test on the shifts in the distribution of the forcing variable [26], we found no evidence of a discontinuity (estimated discontinuity: 0.02, standard error: 0.03).

Table A3 in the Supplementary material reports the results from a formal test of shifts in slopes at the kink point. The estimates correspond to our main specification but with the covariates as the outcome variables. We found statistically significant slope changes for most covariates. These were included in Eq. 5 in levels and interacted with the dummy variable *D* to allow the effect of these covariates to vary at the kink point [cf. 17].

A specific concern is that a patient who maximizes dynamic utility will take into account that a purchase today will reduce the price of a potential purchase in the future [27]. This may cause an upward bias in price elasticity estimates around the kink. We address this concern by conducting a robustness check in which patients only at the very end of their fiscal year are included in the sample (as the end of a fiscal year approaches, the likelihood of the current purchase affecting the price of future purchases tends to fall to zero).

Finally and importantly, a common concern about the RKD is the scope for detecting spurious effects driven by nonlinearities in the underlying relationship between the outcome (additional drug purchase) and the running variable (accumulated total costs) [e.g., 19, 20]. In short, the identification of the numerator in Eq. 3 is based on the change in slopes at the threshold (1100 SEK). But, to apply the RKD approach (as is the case with the regression discontinuity approach) it is typically necessary to use observations extending the thresholds to some degree (the bandwidth). Suppose the relationship between the outcome and the running variable is nonlinear. In that case, the estimated slope in each window to the left and right of the threshold will most likely differ from the slopes just at the threshold. This difference will cause a bias in the estimates of the numerator and consequently in the estimate of the price elasticity. We expand and graphically demonstrate this problem further in the supplementary material (see Fig. A3).

Solutions suggested to account for confounding due to nonlinearities around the threshold include using higher-order polynomials to explicitly model the nonlinearities. However, this causes other concerns, such as noisy estimates and poor coverage of confidence intervals [24]. Another suggested solution is to apply permutation tests using placebo kinks at varying locations in the forcing variable. However, this still leaves room for the concern that there is a smooth nonlinear relationship specifically at the kink used to identify the causal effect. To overcome this, Landais [18] suggested implementing a double-difference RKD whenever there is a policy change that shifts the location of the kink.

### Double-difference RKD

The opportunity to implement a double-difference (DD) RKD comes from the reform in January 2012, when the first cost-sharing threshold was raised from 900 to 1100 SEK. The DD-RKD compares an estimate of the numerator in Eq. 3 at the 1100 threshold using data from 2012 to 2013 (when the threshold was in place) with an estimate of the numerator using data from 2010 to 2011 (with no threshold in place). This means that the numerator of the RKD estimator in the DD-RKD is the difference in the estimated numerator in the 2012–13 data with the numerator in 2010–11 subtracted. Suppose there are no nonlinearities at the cost threshold of SEK 1100. In that case, there should be no estimated effect using the 2010–11 data (the “placebo test”), thus leaving the estimated numerator from the 2012–13 period unaffected. Adopting a DD-RKD approach does not imply that we hypothesize a specific nonlinear relationship around the kink. Still, it allows us to rule out that potentially identified effects in the standard RKD are not driven by spurious nonlinearities around the kink. Specifically, we estimate:

6 $$ \begin{aligned} E\left[ {Y|V = v} \right] =\,\, & \mu_{0} + \mathop \sum \limits_{p = 1}^{{\overline{p}}} \omega_{p} \left( {v - k} \right)^{p} + \beta_{p} \left( {v - k} \right)^{p} D \\ & + \gamma Post + \eta_{p} \left( {v - k} \right)^{p} Post + \delta_{p} \left( {v - k} \right)^{p} DPost, \\ \end{aligned} $$

where ${\delta }_{1}$ is the coefficient of interest, which shows the magnitude of the change in the slope at the kink in 2012–13, controlling for any potential effect at the same kink in 2010–11.

## Results

### RKD results

**Fig. 3:**
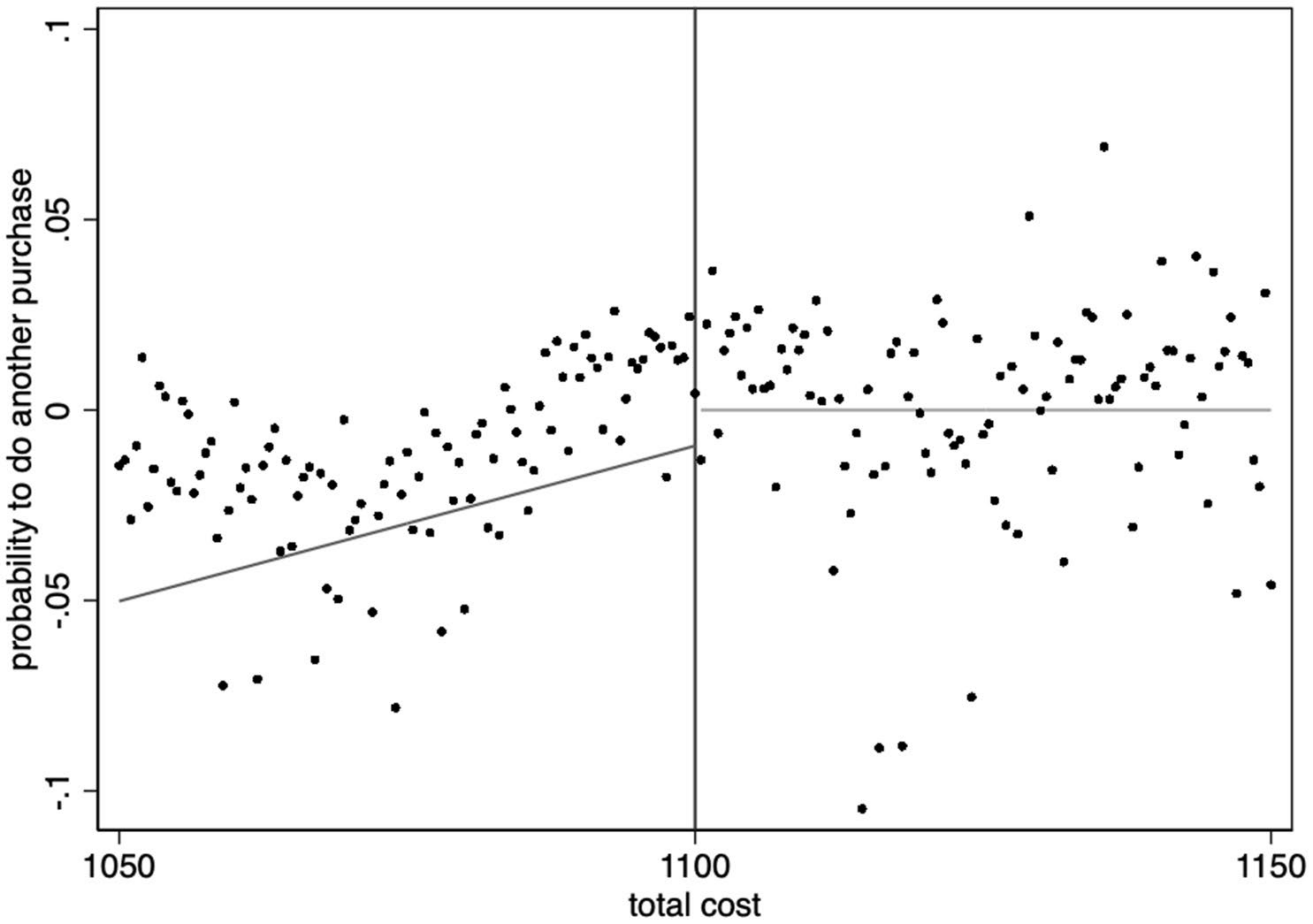
Probability of another drug purchase at cost threshold. Each dot represents the probability of another purchase at varying total costs (normalized for the overall trend between total costs and the absolute probability of another purchase in 2012–2013)

Figure 3 shows the relationship between the probability of another drug purchase and accumulated costs around the threshold by plotting the average purchase propensity in one-krona (SEK 1) intervals and the associated fitted lines on both sides of the threshold using data from 2012 to 13. A clear kink in purchase propensity is identified: to the left of the kink, where consumers face decreasing prices, the probability of additional purchase increases when approaching the kink. To the right of the kink, where consumers face constant prices, the probability of making another drug purchase is roughly constant.

Table 1 shows the estimated elasticities computed separately for the years 2010–11 (“placebo period”) and 2012–13 (“treatment period”) with different bandwidths around the SEK 1100 threshold. Control variables include the number of weeks since the beginning of the fiscal year (and its square), sex, household income, a dummy for higher educational attainment, and age. To allow for kinks in control variables, all controls are interacted with a dummy for all observations above the threshold.

**Table 1:** RKD estimates of demand elasticities

Bandwidth	Coefficient (std. err.)	Elasticity	Elasticity 95% CI	*N*
Treatment period (2012–13)				
50	− 0.0007 (0.0001)	− 0.19	[− 0.26 to − 0.12]	127,604
40	− 0.0005 (0.0001)	− 0.15	[− 0.25 to − 0.05]	102,189
30	− 0.001 (0.0002)	− 0.28	[− 0.40 to − 0.16]	77,809
Placebo period (2010–11)				
50	− 0.0004 (0.0001)	− 0.10	[− 0.13 to − 0.05]	127,500
40	− 0.0008 (0.0001)	− 0.18	[− 0.24 to − 0.11]	101,342
30	− 0.0013 (0.0002)	− 0.29	[− 0.38 to − 0.20]	76,878

Results for a first-order polynomial. Controls: number of weeks since the beginning of the fiscal year (and square), female, household income, higher educational attainment, and age. All controls were also included interacted with a dummy for observations on the right side of the kink. The 95% confidence interval (CI) is based on bias-corrected bootstrapped standard errors

For the “treatment period” (2012–13), all estimated elasticities are negative and statistically different from zero. The estimates vary from approximately − 0.15 to − 0.3 depending on the bandwidth considered. As expected, the precision of the estimates improves with bandwidth and hence sample size. However, the estimated elasticities for the “placebo period” (2010–11) are also statistically significant and almost identical in magnitude (varying from − 0.1 to − 0.3). These findings suggest confounding nonlinearities around the threshold and that what we detect for 2012–13 is most likely not an actual causal price effect. The sensitivity analyses based on quadratic polynomials and triangular kernels provided similar results (see Table A4 in the Supplementary material).

### Double-difference RKD results

Table 2 reports the DD-RKD estimates, providing an approach to control for potential spurious kinks around the threshold. The main coefficient estimates of interest refer to $\left({v}_{ij}-k\right)D\mathrm{Post}$ from Eq. 6. In line with our expectation based on the results shown in Table 1, we find no statistically significant effects using the DD-RKD approach. The estimated elasticity in the largest bandwidth is − 0.05, with the 95% CI ranging from − 0.12 to 0.02. This indicates that the change in consumption patterns around the threshold is most likely driven by a nonlinear relationship between accumulated total cost and probability of a further drug purchase and that this relationship is unrelated to the shift in the cost-sharing.

**Table 2:** DD-RKD estimates of differences in demand elasticities

Window	Coefficient (std. err.)	Elasticity	Elasticity 95% CI	*N*
50	− 0.0002 (0.0001)	− 0.05	[− 0.12 to 0.02]	255,104
40	0.0003 (0.0002)	0.07	[− 0.02 to 0.17]	203,351
30	0.0003 (0.0003)	0.07	[− 0.06 to 0.21]	154,687

Results for the first-order polynomial. Controls: number of weeks since the beginning of the fiscal year (and square), female, household income, higher educational attainment, and age. To allow for kinks in controls, all controls were interacted with a dummy for observations above the threshold

**Fig. 4:**
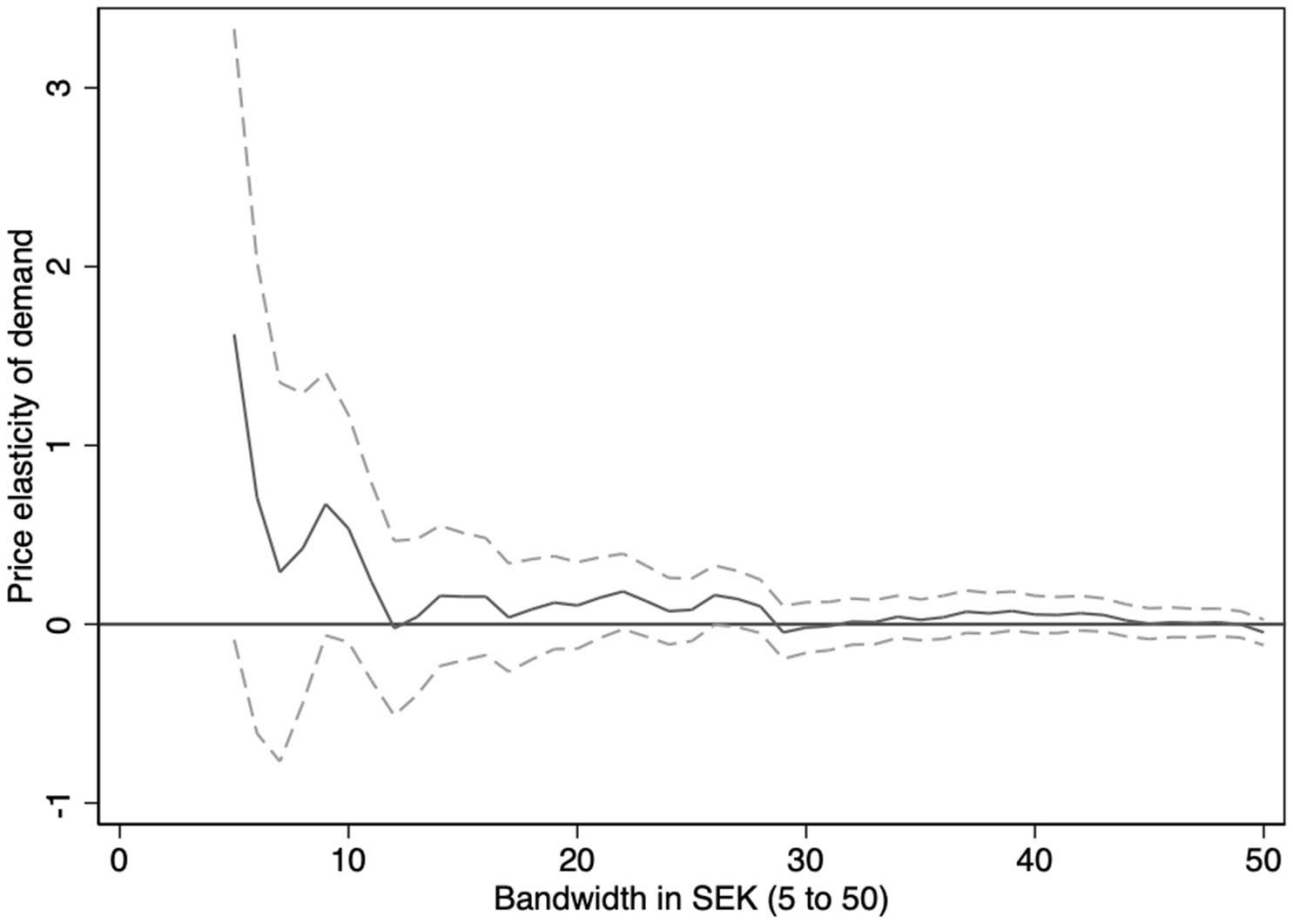
Elasticity estimates based on the DD-RKD estimator over varying bandwidth. The solid line shows the elasticity estimate in each bandwidth (starting at SEK 5 and up to SEK 50), and the dashed lines represent the 95% confidence interval

To verify that the results shown in Table 2 are not due to the specific bandwidths chosen, Fig. 4 shows the elasticity estimates based on separate regression bandwidths, starting at SEK 5 and up to SEK 50 (incrementally increasing the bandwidth by SEK 1). We generally find the estimated elasticity to be close to null, albeit with a large confidence interval in the smaller bandwidths (as expected, considering the reductions in observations as the bandwidth approaches 0).

As additional robustness tests for the DD-RKD, we re-run the models with quadratic polynomials and triangular kernels. Once again, they yield the same qualitative interpretations (see Table A5 in the Supplementary material). We also estimate Eq. 6 using only individuals in the 10th, 11th, and 12th months of the fiscal year. This reduces potential confounding due to dynamic optimization that might create an upward bias of our previous elasticity estimates. For a patient in the last month of its fiscal year, the future price effects of a current purchase decision will last only a few weeks. The results (Table A6 in the Supplementary material) are in line with those seen in Table 2, i.e., we cannot reject the hypothesis that price has no causal effect (although the confidence intervals are much wider, as expected, given a substantial reduction in sample size).

### Double-difference RKD results: testing for heterogeneity

We also implement the DD-RKD in sub-groups where we split the sample across sexes, age groups, and income quartiles (see Table A7, Supplementary material). In conclusion, there are no indications that there is a large or statistically significant price elasticity of demand across the different sub-groups. The elasticity point estimates range from − 0.15 to 0.07, and the 95% CI for all point estimates overlaps a null effect.

## Concluding discussion

Using drug-purchasing records for 2010–13 from a random sample of 400,000 Swedes, we measured the effect of different levels of cost-sharing on prescription drug purchases by exploiting the kinked reimbursement scheme. In the standard RKD approach, elasticities ranged from − 0.15 to − 0.3. These estimates are similar to results previously reported when the RKD and a similar kinked reimbursement scheme were used in Denmark [17]. Additionally, we were able to perform a DD-RKD by taking advantage of the fact that the threshold for cost-sharing changed on January 1, 2012 (an upward shift of SEK 200 in all thresholds for the cost-sharing). If the effect on demand were due to the kink in the reimbursement scheme, we would expect no effect in 2010–11, when there was no change in the cost-sharing at the threshold considered. To the best of our knowledge, we are the first to apply a DD-RKD in the context of demand for health care. The analysis conducted for 2010–11 showed very similar coefficients, and similar elasticities, to those estimated for 2012–13. Thus, our results for a full regression model, including the data from both the placebo period and the treatment period in a DD-RKD setting, failed to reject the null hypothesis of no price sensitivity. The elasticities estimated were also close to null. They were estimated with good precision in the larger bandwidths, indicating a reasonable probability for rejecting a price sensitivity of any major magnitude. For example, calculating the ex-post Minimal Detectable Effect (MDE) with a 5% level of statistical significance and 80% power level [28], our results (Table 2, largest window) indicate that we would be able to detect a significant price elasticity smaller than − 0.10 (or expressed in absolute terms, larger than 0.1). In the smaller windows around the kink the MDE becomes larger (0.13 and 0.19).

It has been suggested that the sensitivity of the RKD approach to nonlinearities around the threshold could be solved by using a very small bandwidth around the kink or by higher-order polynomials [19]. However, the drawback of these approaches is that it quickly reduces the statistical power, as shown in Fig. 4 when considering smaller bandwidths. The DD-RKD approach is, thus, an important improvement regarding the validity of the RKD causal inference approach.

However, it should clearly be stated that even though the 95% confidence intervals indicate no major effect of the cost-sharing, the failure to reject the null should not be interpreted as that the effect is specifically null.

Given the rising drug costs seen in almost all OECD countries, the use of cost-sharing mechanisms has been exploited by governments as a way to reduce pressure on their budgets and reduce moral hazards. Our results suggest that changes in cost-sharing with small absolute monetary consequences may not restrict prescription drug costs very much, at least not in a system where the cost-sharing level is a continuous piecewise linear function of the accumulated cost of prescribed cost drugs. Additionally, our results cannot elucidate substantial changes in out-of-pocket costs, and therefore obviously do not rule out cost containment from substantial policy changes. From a policy perspective, our results indicate that deciding at what thresholds cost-sharing starts may not be very important when the maximum out-of-pocket payment is low.

## Supplementary Information

Below is the link to the electronic supplementary material.

Supplementary file1 (DOCX 2339 KB)

## Data Availability

Data are proprietary, but can be accessed from the respective government authorities upon ethical approval.

## Footnotes

1 The use of the RKD might be found also in other contexts: for example, the discontinuities in government unemployment insurance payments in Louisiana and Missouri have been exploited by Landais [18] and Card et al. [22], respectively, to analyze how long people spend in unemployment as function of the amount of benefit they receive.

2 The price of the typical prescription drug is around SEK 100 (11 USD). An individual with an accumulated total cost of SEK 1080 would pay SEK 60 (20 + 0.5 $\times$ 80) for the drug. An individual with an accumulated total cost of SEK 1120 would pay SEK 50 (0.5 $\times$ 100) for the same prescription.

3 We consider individuals only at the first point in time within each fiscal year in which their total costs fall within the selected bandwidth around the kink. Similarly, the average price of the purchase ($\overline{\mathrm{price} }$) and the propensity to purchase another drug in the following month ($\overline{\mathrm{quantity} }$) are computed only for observations that fall within the considered bandwidth considered.

## Code availability

Available upon request.
