# Supplementary material for: The impact of cost-sharing on prescription drug demand: evidence from a double-difference regression kink design
Source: Eur J Health Econ. 2022 Feb 25;23(9):1591–9. doi: 10.1007/s10198-022-01446-w (PMC9666319; doi:10.1007/s10198-022-01446-w)
Supplement: Supplementary file 1 — Supplementary file1 (DOCX 2339 KB) [file 10198_2022_1446_MOESM1_ESM.docx]

**Supplementary Material – Table of Content**

[Policy Setting and Data ii](#_Toc89845197)

[Table A1. Sample summary statistics: mean (std.dev.) ii](#_Toc89845198)

[Table A2. Sample summary statistics: annual prescription drug expenditures iii](#_Toc89845199)

[Figure A1. Histogram of total costs (main sample) iv](#_Toc89845200)

[Empirical Approach v](#_Toc89845201)

[Figure A2. Distribution of observations and covariates around the kink v](#_Toc89845202)

[Table A3. Shifts in slopes for covariates vi](#_Toc89845203)

[Figure A3. Bias in the Regression Kink Design due to nonlinearities around the kink vii](#_Toc89845204)

[Table A4. RKD estimates using quadratic polynomials and triangular kernels: elasticities and 95% CI in brackets viii](#_Toc89845205)

[Table A5. DD-RKD estimates using quadratic polynomials and triangular kernels: elasticities and 95% CI in brackets ix](#_Toc89845206)

[Table A6. Diff-RKD estimates after minimizing the influence from dynamic optimization x](#_Toc89845207)

# Policy Setting and Data

## Table A1. Sample summary statistics: mean (std.dev.)

|  | Main sample | Analysis sample |
| --- | --- | --- |
| Age | 49  (19) | 62  (19) |
| Woman | 0.50 | 0.62 |
| Married | 0.43 | 0.46 |
| University education | 0.38 | 0.25 |
| Employed | 0.59 | 0.37 |
| Annual after-tax income in SEK (individual) | 211,118  (536,569) | 206,325  (393,322) |
| Annual after-tax income in SEK (household) | 386,484  (713,901) | 349,293  (851,459) |

**Comment:** The main sample is the individuals with any prescription drug costs over the years 2010-2013 out of the 400,000 randomly sampled Sweden. The analysis sample is the individuals that at any time during the fiscal year enters into and/or thru the window around the first kink. One Swedish Krona (SEK) is approx. 0.125 USD.

## Table A2. Sample summary statistics: annual prescription drug expenditures

|  | Main sample | | Analysis sample | |
| --- | --- | --- | --- | --- |
|  | Mean  (std. dev) | Median  (IQR) | Mean  (std. dev) | Median  (IQR) |
| Annual total costs | 3,337  (20,466) | 554  (0 – 2,314) | 4,800  (13,442) | 2,429  (1,487 – 4,766) |
| Annual out of pocket costs | 563  (1,655) | 288  (0 – 855) | 938  (260) | 1016  (977 – 1074) |

**Comment:** The table gives summary statistics for the main sample of 400,000 randomly selected Swedes (in 2010) aged 18 and above (over the years 2010-2013). The analysis sample is the individuals that enter into the bandwidth and is used for the analyses. One Swedish Krona (SEK) is approx. 0.125 USD.

## Figure A1. Histogram of total costs (main sample)


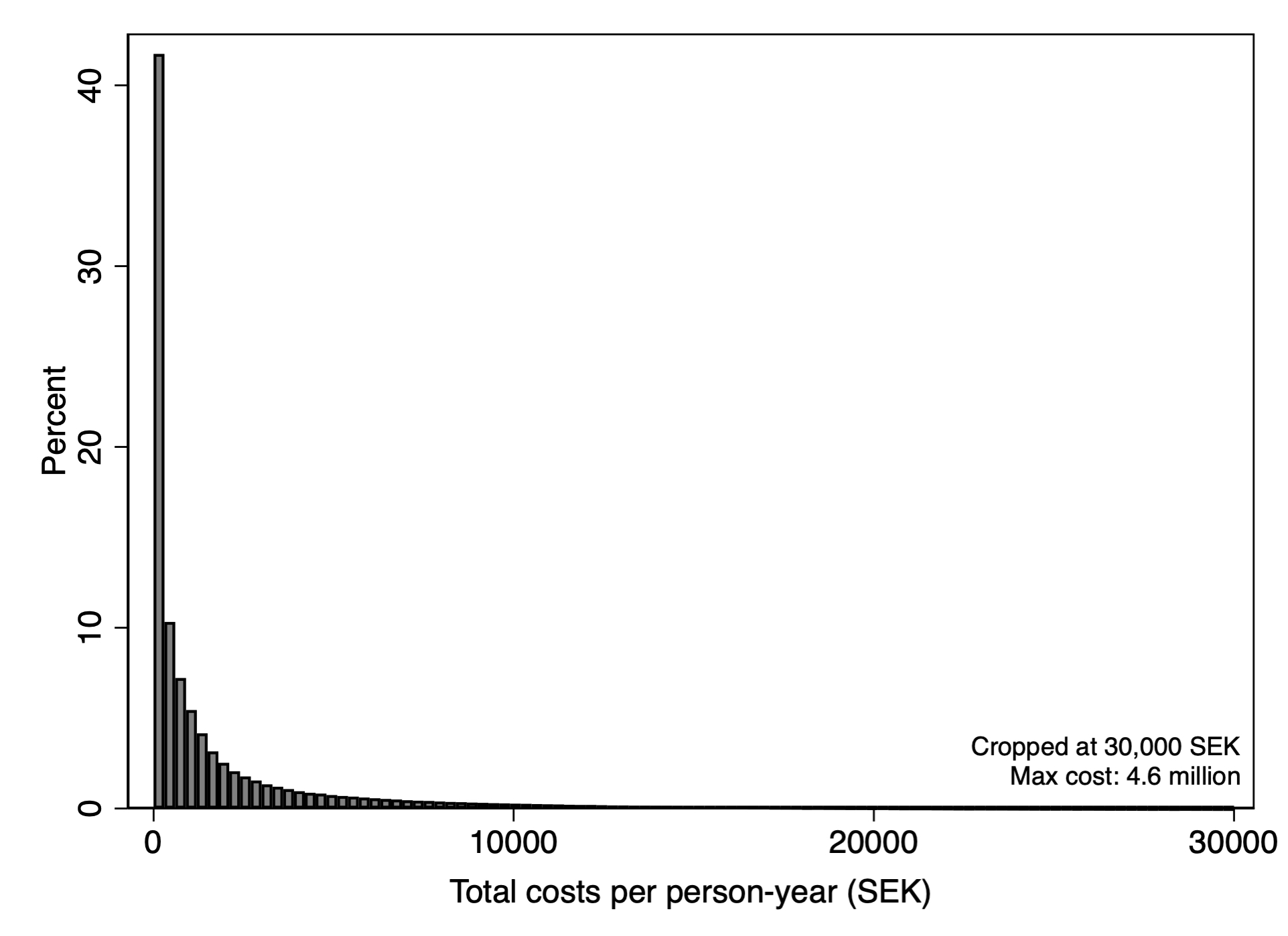


**Comment:** The histogram shows the distribution of total prescription drug costs per person-year in the main sample (1,560,341 person-year observations). Each bin represents 300 SEK. Around 29% of the person-year observations have no costs at all.

# Empirical Approach

## Figure A2. Distribution of observations and covariates around the kink


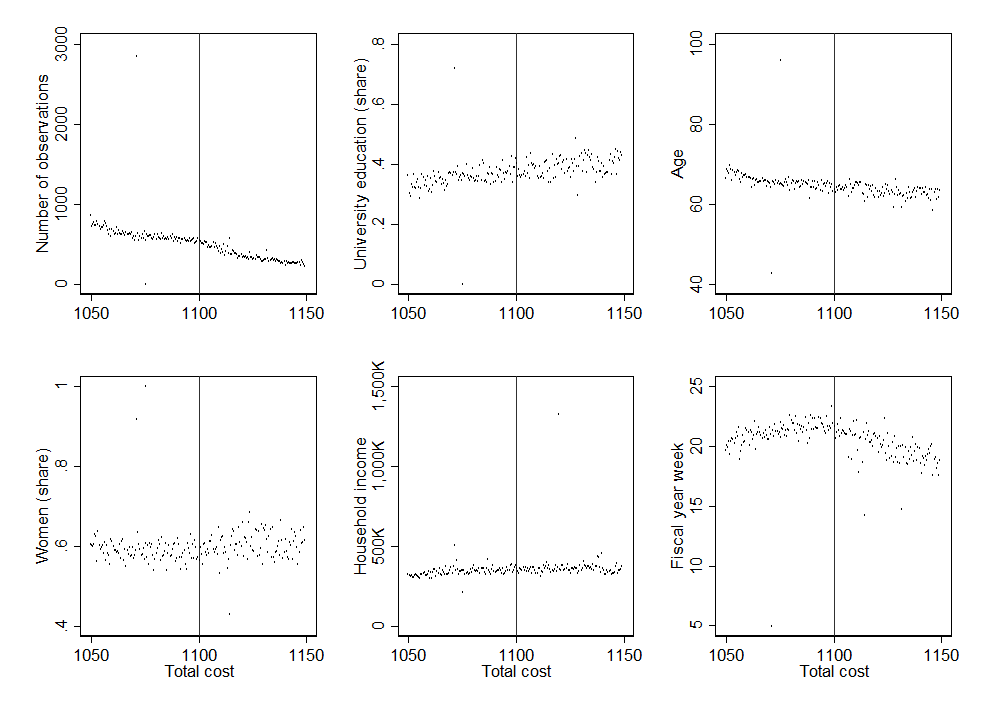


**Note:** Frequency distribution of observations, and covariate values around the kink. Linear parametric specification with a bandwidth of 50 SEK. Results are similar for other specifications and windows.

## Table A3. Shifts in slopes for covariates

|  | Coefficient | p-value |
| --- | --- | --- |
| Household income | −690 | 0.205 |
| Low education | 0.001 | 0.000 |
| Age | 0.025 | 0.001 |
| Female | 0.001 | 0.000 |
| Weeks since beginning | −0.098 | 0.000 |

**Note:** Linear parametric specification for the two largest windows around the kink are presented here. Window = 50 SEK. Results are similar for other specifications and windows.

## Figure A3. Bias in the Regression Kink Design due to nonlinearities around the kink

Propensity to purchase a drug (Y)

e

f

d

$$-\hat{\beta_{1}}$$

B

c

A

Total cost (v)

1100 SEK

+h

-h

**Note:** This description is based on Ando [19, p.1292]. The figure shows a hypothetical nonlinear relationship between the probability of an additional drug purchase and the accumulated total cost from point A to B that is increasing at a declining rate to the left of the threshold and flat to the right of the threshold. The true kink at the threshold (V=1100 SEK) is null, since the slope is identical at both sides of the threshold at V=1100, i.e.there is no effect of the kink (change in cost-sharing) per se on the probability of an additional drug purchase. However, when applying the RKD it is typically necessary to use a bandwidth (-h to +h) that extends away from the threshold (in our approach we use varying bandwidths with a window size between 5 and 50 SEK). The dashed *cd* line is the linear fit to the left of the threshold. The counterfactual to the right of the threshold is the *de* line, which will be compared to a fitted horizontal line (*df*). The estimated change in slope at the kink will be $-\hat{\beta_{1}}$, indicating a substantial effect in the outcome variable, which is driven by the nonlinear relationship that is not properly addressed in the local linear regression. This highlight that Eq.3 in the main text is valid only exactly at the kink/threshold.

## Table A4. RKD estimates using quadratic polynomials and triangular kernels: elasticities and 95% CI in brackets

| **Window** | **Quadratic polynomials** | | **Triangular kernel** | **Quadratic polynomials and triangular kernel** |
| --- | --- | --- | --- | --- |
| **Treated period (2012–2013)** | | | | |
| 50 | -0.12  (-0.36 to 0.11) |  | -0.17  (-0.24 to -0.09) | -0.30  (-0.58 to -0.01) |
| 40 | -0.45  (-0.76 to -0.14) |  | -0.18  (-0.27 to -0.08) | -0.36  (-0.72 to -0.01) |
| 30 | 0.02  (-0.46 to 0.50) |  | -0.26  (-0.38 to -0.14) | 0.06  (-0.41 to 0.54) |
| **Placebo period (2010–2011)** | | | | |
| 50 | -0.55  (-0.71 to -0.38) |  | -0.19  (-0.24 to -0.14) | -0.51  (-0.70 to -0.32) |
| 40 | -0.60  (-0.83 to -0.38) |  | -0.22  (-0.29 to -0.16) | -0.53  (-0.77 to -0.29) |
| 30 | -0.34  (-0.68 to 0.01) |  | -0.38  (-0.38 to -0.21) | -0.26  (-0.58 to 0.06) |

**Note:** Controls: number of weeks since the beginning of the fiscal year (and square), female, household income, university education, and age. To allow for kinks in controls, all controls have been interacted with a dummy for being above the threshold.

## Table A5. DD-RKD estimates using quadratic polynomials and triangular kernels: elasticities and 95% CI in brackets

| **Window** | **Quadratic polynomials** | **Triangular kernel** | **Quadratic polynomials and triangular kernel** |
| --- | --- | --- | --- |
| 50 | -0.05  (-0.12 to 0.02) | 0.06  (-0.03 to 0.15) | 0.06  (-0.03 to 0.15) |
| 40 | 0.08  (-0.03 to 0.18) | 0.09  (-0.02 to 0.20) | 0.09  (-0.01 to 0.20) |
| 30 | 0.07  (-0.07 to 0.21) | 0.09  (-0.05 to 0.23) | 0.09  (-0.06 to 0.23) |

**Note:** Controls: number of weeks since the beginning of the fiscal year (and square), female, household income, university education, age. To allow for kinks in controls, all controls have been interacted with a dummy for being above the threshold.

## Table A6. Diff-RKD estimates after minimizing the influence from dynamic optimization

| **Fiscal year** | **Elasticity** | **Elasticity 95% CI** | **N** |
| --- | --- | --- | --- |
| Last 3 months only | 0.11 | −0.15 to 0.28 | 42,184 |
| Last 2 months only | 0.06 | −0.23 to 0.46 | 27,157 |
| Last month | 0.25 | −0.46 to 0.98 | 13,855 |

**Note:** Results for a first-order polynomial. Controls: female, household income, university education, age. To allow for kinks in controls, all controls have been interacted with a dummy for being above the threshold.

*Table A7. DD-RKD estimates in different sub-groups (“Heterogeneity analyses”)*

| **Window** | **Elasticity** | **Elasticity 95% CI** | **N** |
| --- | --- | --- | --- |
| Women | −0.004 | [−0.09 to 0.08] | 155,548 |
| Men | -0.12 | [−0.25 to 0.01] | 99,555 |
| Age <45 | 0.07 | [−0.14 to 0.36] | 44,244 |
| Age 45-65 | -0.15 | [−0.29 to -0.01] | 84,604 |
| Age 65+ | -0.06 | [−0.14 to 0.02] | 126,275 |
| Income Q1 | 0.01 | [−0.12 to 0.14] | 63,777 |
| Income Q2 | -0.05 | [−0.15 to 0.05] | 63,777 |
| Income Q3 | 0.01 | [−0.11 to 0.13] | 63,774 |
| Income Q4 | -0.12 | [−0.26 to 0.01] | 63,775 |

**Note:** Results for the first-order polynomial with window=50. Controls: number of weeks since the beginning of the fiscal year (and square), female, household income, higher educational attainment, and age. To allow for kinks in controls, all controls were interacted with a dummy for observations above the threshold.
